# Mutational signatures for breast cancer diagnosis using artificial intelligence

**DOI:** 10.1186/s43046-023-00173-4

**Published:** 2023-05-15

**Authors:** Patrick Odhiambo, Harrison Okello, Annette Wakaanya, Clabe Wekesa, Patrick Okoth

**Affiliations:** 1 Department of Biological Sciences, School of Natural and Applied Sciences, Masinde Muliro University of Science and Technology, P.O. Box 190, Kakamega, 50100 Kenya; 2 Department of Mathematics, School of Natural and Applied Sciences, Masinde Muliro University of Science and Technology, P.O. Box 190, Kakamega, 50100 Kenya

**Keywords:** Breast cancer, Somatic mutations, Early diagnosis, Machine learning, Artificial intelligence

## Abstract

**Background:**

Breast cancer is the most common female cancer worldwide. Its diagnosis and prognosis remain scanty, imprecise, and poorly documented. Previous studies have indicated that some genetic mutational signatures are suspected to lead to progression of various breast cancer scenarios. There is paucity of data on the role of AI tools in delineating breast cancer mutational signatures. This study sought to investigate the relationship between breast cancer genetic mutational profiles using artificial intelligence models with a view to developing an accurate prognostic prediction based on breast cancer genetic signatures. Prior research on breast cancer has been based on symptoms, origin, and tumor size. It has not been investigated whether diagnosis of breast cancer can be made utilizing AI platforms like Cytoscape, Phenolyzer, and Geneshot with potential for better prognostic power. This is the first ever attempt for a combinatorial approach to breast cancer diagnosis using different AI platforms.

**Method:**

Artificial intelligence (AI) are mathematical algorithms that simulate human cognitive abilities and solve difficult healthcare issues such as complicated biological abnormalities like those experienced in breast cancer scenarios. The current models aimed to predict outcomes and prognosis by correlating imaging phenotypes with genetic mutations, tumor profiles, and hormone receptor status and development of imaging biomarkers that combine tumor and patient-specific features. Geneshotsav 2021, Cytoscape 3.9.1, and Phenolyzer Nature Methods, 12:841–843 (2015) tools, were used to mine breast cancer-associated mutational signatures and provided useful alternative computational tools for discerning pathways and enriched networks of genes of similarity with the overall goal of providing a systematic view of the variety of mutational processes that lead to breast cancer development. The development of novel-tailored pharmaceuticals, as well as the distribution of prospective treatment alternatives, would be aided by the collection of massive datasets and the use of such tools as diagnostic markers.

**Results:**

Specific DNA-maintenance defects, endogenous or environmental exposures, and cancer genomic signatures are connected. The PubMed database (Geneshot) search for the keywords yielded a total of 21,921 genes associated with breast cancer. Then, based on their propensity to result in gene mutations, the genes were screened using the Phenolyzer software. These platforms lend credence to the fact that breast cancer diagnosis using Cytoscape 3.9.1, Phenolyzer, and Geneshot 2021 reveals high profile of the following mutational signatures: BRCA1, BRCA2, TP53, CHEK2, PTEN, CDH1, BRIP1, RAD51C, CASP3, CREBBP, and SMAD3.

## Background

Breast cancer is the most common type of cancer in women [1]. Breast cancer develops when the breast’s normal cell tissues divide abnormally and uncontrollably. These abnormal cells accumulate into a mass of tissue that eventually turns into a tumor [2]. Breast abnormalities can, however, be challenging to diagnose. Traditionally, breast cancer diagnosis and treatment have been based on clinical symptoms, tumor shape, and site of origin [3, 4]. To detect breast cancer, many technologies have been developed, including mammography, ultrasound, and thermography [5]. Mammography is the process of using low-energy X-rays to examine the human breast for diagnosis and screening [6]. Since mammography was not very successful for dense breasts, diagnostic models were required [7]. Thermography is a procedure in which a special camera that senses heat is used to record the temperature of the skin that covers the breasts [8]. The most crucial aspect of image-based diagnosis is without a doubt the examination of patient data and expert opinion, but there are numerous other aspects that might influence this kind of diagnosis as well. Image noise, the radiologist’s visual perception skills, inadequate clarity, low contrast, and the radiologists lack of expertise are some of the issues that can impair an image-based diagnosis. In order to diagnose breast cancer, the current study focused on various AI signatures processing techniques that can increase diagnostic accuracy by reducing false positives.

Despite the fact that chemoradiotherapy is the mainstay of care for various cancers, most patients who are initially receptive developed resistance after multiple relapses including platinum resistance [9]. Furthermore, compared to chemoradiotherapy, molecular-targeted therapy is more likely to be successful and less harmful [10]. Through analyses done on cancer genomes, different cancer diseases have been identified through genetic mutations [11]. The discovery of driver gene mutations that provide cancer cells with growth advantages and thus promote tumor genesis has been a major focus of cancer diagnostic research in recent years [4].

A biomarker is a molecule found in blood, other body fluids, or tissues that indicates whether a process is normal or pathological [12]. They may also be used as direct treatment targets. Some biomarkers, such as estrogen, are well known for their importance in mammary gland growth, development, and function, and detecting estrogen receptors (ERs) in breast cancers, along with progesterone receptors, has long been beneficial for prognosis and cancer therapy selection [13–15]. Drug development today faces a slew of issues, including high failure rates due to the lack of preclinical trials and complex clinical outcomes. A new generation of biomarkers must be identified using computational methods for relevant clinical practice and medication discovery to boost the success rate [16]. The fast rising number of medications targeting proteins encoded by mutant driver genes has fuelled the development of tests for reliable mutational identification for breast cancer diagnosis [4]. Each mutational process generates a unique signature that may include genome rearrangements, deletions, insertions, base substitutions, chromosome copy-number changes, DNA damage or modification, DNA repair, and either normal or abnormal DNA replication leading to breast cancer [17, 18]. Despite the fact that this knowledge has advanced drug development and improved cancer therapy, majority of patients have not benefited from this technique due to low response rates to targeted therapies and inappropriate biomarkers. Consequently, greater molecular identification of tumors and accurate biomarkers for patient categorization are required [4].

Artificial intelligence is a field of computer science and engineering focused on the creation of systems that can reason, learn, and act autonomously [19]. Machine learning (ML), a subset of artificial intelligence (AI), creates neural network-based algorithms that allow machines to learn and solve problems similarly to the human brain. Deep learning is a type of machine learning that allows computers to learn from their mistakes and comprehend the world by the use of a hierarchical conceptual structure [20]. It can process data to recognize images, objects, and languages, as well as improve drug development, increase medication precision, improve diagnosis, and assist humans in making clinical decisions.

### Role of artificial intelligence in medicine

Over the last few decades, artificial intelligence (AI) has become increasingly important in the world [21]. Artificial intelligence has impacted on people’s lives in different ways [21]. Using fingerprints for security, signing into email accounts and purchasing items online are all areas where artificial intelligence is being used. The medical industry is another field where use of artificial intelligence is being explored, notably in terms of diagnosis and treatment management [21]. As a result, there is concern that artificial intelligence will one day be able to perform tasks that humans cannot do. In various researches done, artificial intelligence has been demonstrated to enhance human judgment, aid in healthcare decisions, and improve treatment efficiency in the future [21].

Using various cognitive capabilities, artificial intelligence (AI) aids in the development of novel and accurate analytical procedures for medical images [22]. With the increased interest of radiologists and pathologists, AI techniques accelerate productivity from complex medical imaging data, creating conducive environment for implementing medical-based image analysis and making study more intuitive with ability of changing image data into analytical and decision-based information. AI diagnoses medical issues such as cardiovascular irregularities, musculoskeletal injuries, neurological illnesses, and cancer by providing life-changing insights. Furthermore, artificial intelligence has the ability to improve the traditional medical-based imaging process by increasing efficiency, automating workflow, and computing enormous amounts of data. As a result, combining intelligent ways of dealing with images taken from biological features has provided scientists and researchers with statistical significance value.

#### Manufacturing of drugs

Researchers predict future outcomes and the success of employing a treatment by applying machine learning to pharmaceuticals. Researchers discover probable drug side effects and develop alternative components that lower unwanted effects. AI technologies such as next-generation sequencing and precision medicine are applied in the discovery and manufacture of medications [23].

#### Improving medical records

Although data entry has become more accessible in recent years, keeping health records up to date remains a time-consuming task. The medical staff devotes a significant amount of time to updating the records. It saves time and money as understanding and converting data into different forms using machine learning-based handwriting technologies takes a relatively shorter time. When all of the relevant patient information is already on file, clinicians will have more information to understand the patient’s medical condition, resulting to treatment quality.

In the event of an illness, AI-based algorithms have the potential to discover genetic alterations and abnormal protein-interactions early on [24]. Somatic mutations, particularly noncoding mutations, and their role in generating cancer molecular subtypes have not been adequately examined due to the computational models used to evaluate complicated mutational global patterns [25]. Radiologists streamlined and integrated their diagnostic skills by employing artificial intelligence (AI), such as recognizing and stratifying complex patterns in images, clinical translation of tumor phenotype to genotype, and outcome prediction for therapy and prognostic techniques [26].

The most common predictors of breast cancer are the BRCA1 and BRCA2 gene mutations [27]. Recent genome-wide investigations have showed that the identical mutation pattern seen in BRCA1/2-mutant malignancies can be present in other genes [28]. The open-source software platform Cytoscape 3.9.1 with string plug-in integrates, visualizes, and analyzes measurement data in the context of a network to accurately detect the mutational signature linked to breast cancer using targeted gene panels [29, 30]. Unlike prior models that required whole-exome data or whole genome, these models incorporated a likelihood-based metric with machine-learning algorithms to detect the breast cancer signal even with low mutation counts [28]. By allowing panel-based identification of mutational signatures, Cytoscape significantly increases the number of patients who may be considered for breast cancer therapy [28].

Currently, more than 80% of breast cancers are nonmetastatic localized or locally progressed diseases, and patients have no option but to choose the best treatment plan from a variety of options [31]. The importance of mutational signatures in drug development and therapy alternatives cannot be overstated. For primary non-metastatic breast cancer and clinical stage, the most commonly used bioinformatics model, such as a gene regulatory network, was used to classify breast cancer patients as high, intermediate, or low risk [31]. As a result, new prognostic prediction algorithms are still needed to enhance the precision and sensitivity of breast cancer classification. The accumulation of gene mutations has been linked to the progression of several cancer types in the past [32]. Breast cancer, cervical cancer, lung cancer, nasopharyngeal carcinoma, and other cancers have all used somatic mutation signatures to construct prognostic prediction tools [33].

Tools like Cytoscape 3.9.1, Phenolyzer, and Geneshotsav 2021 with better prognostic power were proposed based on bioinformatics models [31]. Despite the fact that these risk stratification techniques were successful in predicting diagnosis, tumors within the same risk groupings had wildly different clinical outcomes [31]. In breast cancer patients on active surveillance, BRCA1 overexpression has been associated with a greater risk of disease progression [31]. Researchers discovered that a model with a 100-gene signature that categorized breast cancer patients into five distinct subgroups with discrete genomic abnormalities and expression patterns outperformed standard Gleason score predictions in predicting diseases with poor diagnosis in another investigation [31]. This showed that genetic diversity is important for breast cancer classification and can have a higher impact on clinical outcomes. A study was conducted to effectively identify breast cancer-related genes, and it was discovered that protein–protein interactions are helpful in disease-gene prediction [34]. It increased its ability to predict disease-related effects by including Cytoscape data in the design of ligand–protein interactions [34].

## Research methods

In recent years, there has been an increasing interest in using machine learning and artificial intelligence for the diagnosis of breast cancer. This is because these techniques can provide a more accurate and reliable diagnosis than traditional methods, such as mammography. There are many different machine learning and artificial intelligence techniques that can be used for the diagnosis of breast cancer. One of the most promising predictors are mutational signatures. Mutational signatures are patterns of mutations that are unique to a particular cancer. By identifying these signatures, it is possible to more accurately diagnose breast cancer.

### Data sources

Data from Phenolyzer (2015), Cytoscape 3.9.1, and known disease-gene connections were obtained. The statistical approaches for assessing breast cancer-related genes, as well as enhanced techniques for identifying disease-related genes, were then presented. Geneshot 2021 was one of the artificial intelligence platforms used for gene listing and normalization as it allows researchers to enter any search terms and to get ranked lists of genes associated with such terms and genes previously linked with the search terms, as well as novel genes associated with the terms based on data integration from several sources [35]. Cytoscape, an open-source software of artificial intelligence for integrating, presenting, and analyzing measurement data in a network setting, was employed and was used to retrieve networks from PubMed for proteins of interest, selection of proteins based on attributes, integration of external data and mapping on a network, laying out the generated networks, performing enrichment analyses, and identifying functional modules using network clustering. Phenolyzer was used in gene prioritization based on disease to aid in the detection of mutational signatures [36].

### Text mining

Breast cancer-related genes were gathered by text mining from the PubMed database and screened to eliminate false positives. The several mechanisms thought to be involved in the establishment of the ectopic breast cancer tissues were revealed through the characterization of the filtered gene set through gene ontology, pathway, and network analyses. The first step in this procedure was to gather data on breast cancer diagnosis on Geneshot. Natural language processing, which is the ability of a computer program to comprehend both spoken and written language, a subset of artificial intelligence, was used to extract the TM data [37]. The TM was developed using publications obtained from the PubMed database. The database was searched using the keywords “mutational signatures of breast cancer genes” [37]. The required articles were retrieved in Extensible Markup Language (XML) format and added to Mendeley software version: 1.19.8 for reference with material enclosed between XML tag pairs to increase data precision. Ma’ayan Lab designed the Geneshot system for TM search, and it comprised of two modules that were essential for understanding TM’s unique and complex genetic characteristics [35]. For each article, the titles and abstract texts were transformed into the PubTator format [38] through the in-house PERL script. Geneshot pipeline was employed for text mining. Geneshot has two modules, gene mention recognition and gene name normalization. Gene mentions were detected through the CRF +  + library, while gene name normalization was deciphered by Genshot.

### Gene normalization and mentions of recognized genes by Geneshot

Gene normalization is the conversion of a textual name into a unique identifier that is then assigned to a DNA sequence on a certain chromosome [37]. The tasks included species assignment and species-specific gene normalization, which is a difficult problem to solve due to gene mention variance, orthologous gene ambiguity, and intraspecies gene ambiguity [39]. During the gene normalization step, the Geneshot system was utilized to choose novel genes out of a pool of potential reference genes. Gene names were thus determined by context and demand. It is difficult to identify all the genes due to their number. Finding a gene name through TM is time intensive and comparable to searching for a name in a publication, a process known as name entity recognition, which has alarmed researchers [37]. In recent years, techniques for nucleotide excision and repair have improved and evolved. Nucleotide excision repair work is complicated by ongoing gene evolution and frequent renaming of previously known genes, which makes biological domain study difficult [37, 40]. For the gene mention and Geneshot system, the String app was then utilized as a plug-in.

### Candidate genes prioritization by Phenolyzer

The Phenolyzer of Nature Methods, 12:841–843.2015 platforms to be used in gene prioritization across the human genome based on any disease/phenotype terms entered. It has the ability to rank genes in order of importance [37]. The rank is determined by a score representing the possibility rating of the candidate gene, which is highly influenced by the frequency of the gene and the number of candidate genes under investigation [37]. This study include all genes with a score of 0.3.

### Gene Ontology, pathways, and networks analysis by Cytoscape

Gene Ontology is a bioinformatics technique for standardizing genes and gene products expression across species [37]. The main goals of Gene Ontology were to keep a common lexicon for genes and their products up to date, expand it, annotate, integrate, and disseminate the gene and its products, including their properties, and to make the tools for accessing the project’s data as easy to use as possible [16]. The Gene Ontology for the functional interpretation of experimental was done to facilitate enrichment analysis. Gene Ontology is a conceptual- and classification-based biological model framework for describing gene functions. Bingo ver. 2.3 software with GOSlim database was then used in the Gene Ontology analysis [41]. A hypergeometric test followed by customized Bonferroni multiple test correction was performed in order to test for the enrichment. The *p*-value, adjusted to 0.005, was used as significant threshold to identify the enriched GO terms. The R package word cloud was employed in the generation of the word cloud. The pathway enrichment analysis was performed by the David tool [42]. The network and pathways were built with the STRING database 10.5 and then viewed and analyzed with the Cytoscape software 3.9.1

Artificial intelligence tools were utilized to collect and analyze data from breast cancer patients’ genetic profiles. This was done by using machine learning algorithms to identify patterns in the data that may be indicative of a mutational signature. Once the signature was identified, it was used to diagnose breast cancer patients. The data on most affected genes were mined using Geneshot and then used to determine some of the molecular pathways of breast cancer. Applying the disease name in the search bar helped in finding mutational signatures. The results were then filtered by cancer type, mutational signature, and/or gene after selecting the mutation category. A mutational signature for “breast cancer” was seen. Results were then viewed to discover which genes belong to the chosen mutational signature.

Some of the steps in developing a more accurate predictive model for diagnosing breast cancer using artificial intelligence could include the following: larger and more diverse dataset used for training the model. Machine learning that is more sophisticated is specifically designed for dealing with medical data. Domain knowledge was incorporated about breast cancer into the model. The model’s parameter was carefully tuned to optimize its performance**.**

### Associations of disease and genes

**Fig. 1:**
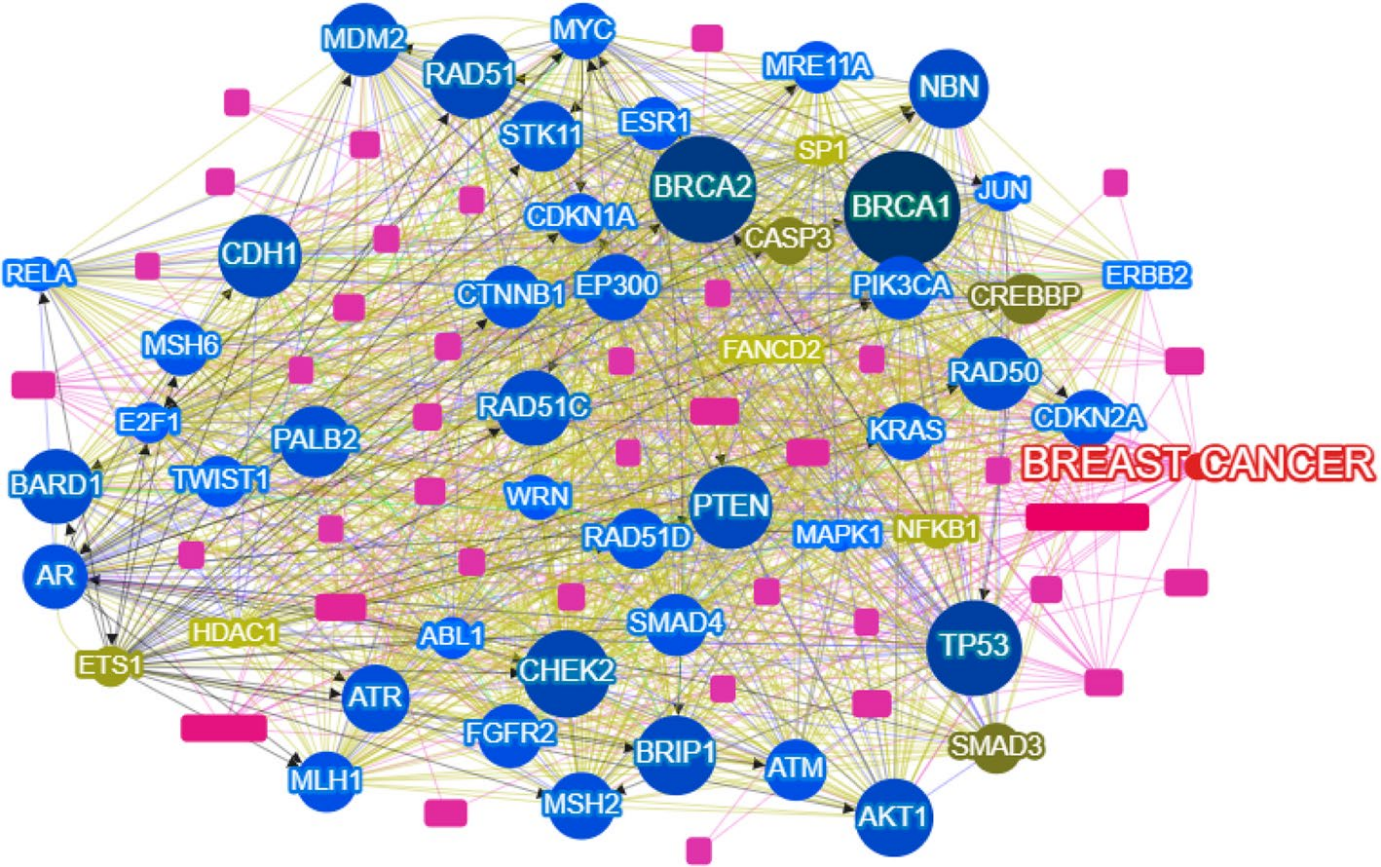
This is a force network of breast cancer-related candidate and novel genes interaction. Seed genes are shown by deep blue circles, while novel genes are represented as green circles

Genes related with disease were found in the Geneshot catalog (https://academic.oup.com/nar/advancearticle/doi/10.1093/nar/gkz393/5494749) [35] and Online Mendelian Inheritance in Man [10, 43]. Some Geneshot disease categories are closely linked, yet scientists classify them differently because some of them have a high percentage of overlapping genes. It was important to group set of genes that were linked. These genes were grouped together using a hierarchical virtual clustering based on their common disease-related genes (Fig. 1).

### Protein–protein interactions (PPIs)

Among several types of data that have been used to predict disease genes, protein–protein interactions were the most extensively used. The PPI network was created using the STRING database (https://string-db.org) [44], which quantifies a variety of studies and interaction types. Only the undirected and weighted networks were considered in this study.

### Data on the protein–protein interaction network and pathways

For disease-related genes, their network and enrichment were subjected to two techniques of analysis: genes linked to breast cancer prediction, prediction performance using the PPI network, and all known disease-associated genes were used as a training set to find possible breast cancer genes using the Cytoscape pathway. Breast cancer candidate genes include the following: Cytoscape enrichment analysis and literature validation were used to examine potential genes linked to breast cancer [10].

## Results

Network analysis of the breast cancer-related subnetwork and pathway analysis of breast cancer-related genes are examples of breast cancer-related gene analyses. Breast cancer genes are a set of genes involved in the progression of the cancer. Using Phenolyzer texts, 21,291 genes were mined. The text mining results have been filtered. The possibility of a gene being produced by chance was assessed and then corrected [37]. The network was built using 200 genes depending on their score value as illustrated on barplot.

### Breast cancer genes mined

A total of 21,921 genes linked to breast cancer were found after searching the PubMed database for the keywords (from 1956 to 2022). The genes were then filtered by Phenolyzer software depending on their risk of causing mutation [36]. The probability of a gene being created by chance was assessed and then corrected. The word cloud, network, and pathway enrichment were all generated using 200 genes that met the statistical significance criterion.

### Genes linked to breast cancer

Genes are short-length chromosomal DNA fragments where instructions for building proteins are contained. These proteins regulate cell structure and function; any alteration in their structure results in defective growth, giving the cells inappropriate information.. Mutations of the DNA are passed down through generations or develop over time. Some are benign, while others have the potential to cause cancer. There were 21,921 found when the term “breast cancer” was searched in the PubMed database. Furthermore, it was revealed that the number of studies published on breast cancer genetic processes has been gradually increasing [37].

The network (Fig. 1) shows force network generated by Phenolyzer (Nature Methods, 12:841–843 2015) showing the interaction of seed and novel genes of breast cancer.

A report summary of mined genes is displayed using Phenolyzer’s barplot to highlight how seed genes and novel genes were significantly organized based on their score values. The higher the score, the more likely a mutation will occur.

**Fig. 2:**
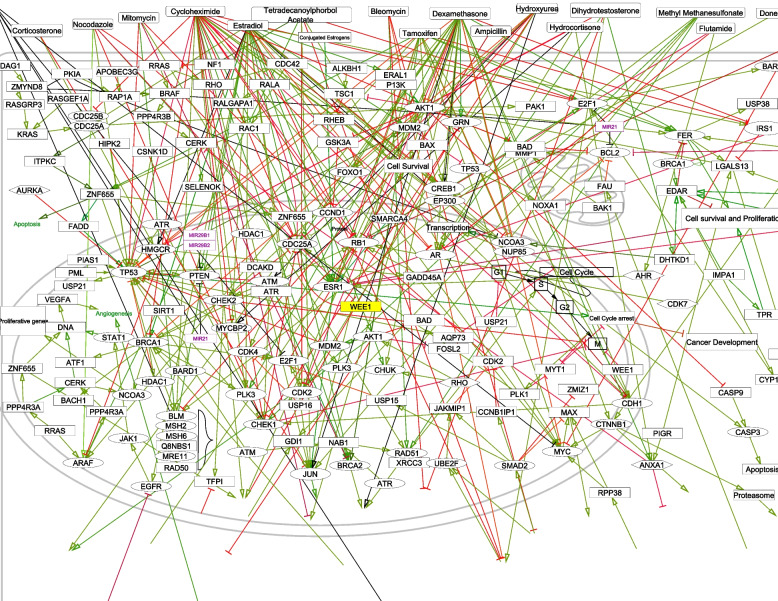
Most breast cancer-related genes have an integrated route and network structure that are affected by particular medicines. The nodes represent the genes created with Cytoscape 3.9.1, whereas the edges represent interactions between genes and already discovered drugs. It is critical to comprehend how existing treatments are linked to the characteristics of mutations, as well as the genes that cause cancer but for which no drugs have been discovered

The interaction between seed genes with currently available breast cancer treatments is depicted in the diagram below (Fig. 2). The networks also show novel genes for which no medicines have been found. Pharmaceutical companies can utilize this information to develop medications to combat breast cancer mutations.

### BReast CAncer gene1 and BReast CAncer gene 2

**Fig. 3:**
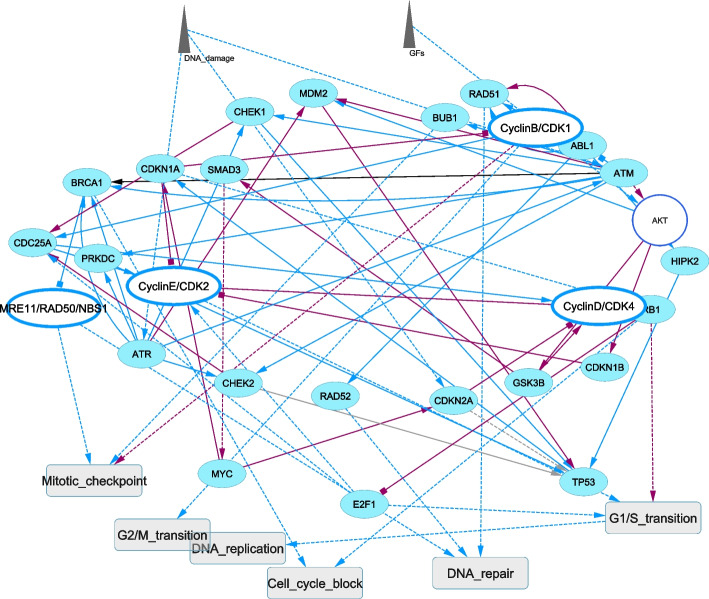
Cytoscape 3.9.1 was used to create a network-based categorization of BRCA1. It identified some of the genes linked to BRCA

Mutations in the BRCA1 and BRCA2 genes cause hereditary breast cancer, which contributes for approximately 3% of all breast cancer cases (roughly 7500 women each year) [45, 46]. These genes, in most situations, prevent particular tumors from occurring. However, some mutations in them may affect their performance, raising the risk of breast cancer if one of these mutations is inherited. If one parent’s BRCA1 or BRCA2 gene is mutated, but the other parent’s BRCA1 or BRCA2 gene is normal, they are inherited in duplicate. When a second mutation in the BRCA1 or BRCA2 gene arises, the normal copy of the gene is damaged and rendered useless. The second mutation, unlike the inherited BRCA1 and BRCA2 mutations, would only be seen in cancer tissue and not throughout the body. The image below displays the association of BRCA1 with other genes generated by Cytoscape version 3.9.1, through which novel genes connected to BRCA1 were predicted as breast cancer mutational signatures that are beneficial in breast cancer diagnosis (Fig. 3).

The image below displays the association of BRCA1 with other genes generated by Cytoscape version 3.9.1, through which novel genes connected to BRCA1 were predicted as breast cancer mutational signatures that are beneficial in breast cancer diagnosis.

Breast cancer type 1 susceptibility protein is an E3 ubiquitin-protein ligase that enhances cellular responses to DNA damage by forming “Lys-6”-linked polyubiquitin chains. It is unclear if it is also involved in polyubiquitin chain creation [47]. E3 ubiquitin-protein ligase’s tumor-suppressing action is critical. The BRCA1-BARD1 heterodimer integrates a wide range of biological activities to maintain genomic stability, including DNA damage repair, ubiquitination, and transcriptional regulation of centrosome microtubules [48]. This protein is required for normal cell cycle progression from G2 to mitosis in both the S-phase and G2-phase of the cell cycle after ionizing irradiation [34]. It also prevents inactive phosphorylated ACACA from being dephosphorylated, therefore inhibiting lipid formation, contributes to homologous recombination repair (HRR), and fine-tunes recombination repair in part by changing PALB2-dependent loading of BRCA2-RAD51 repair machinery at DNA which breaks through direct contact with PALB2 [49].

### TP53 protein and its associated genes

**Fig. 4:**
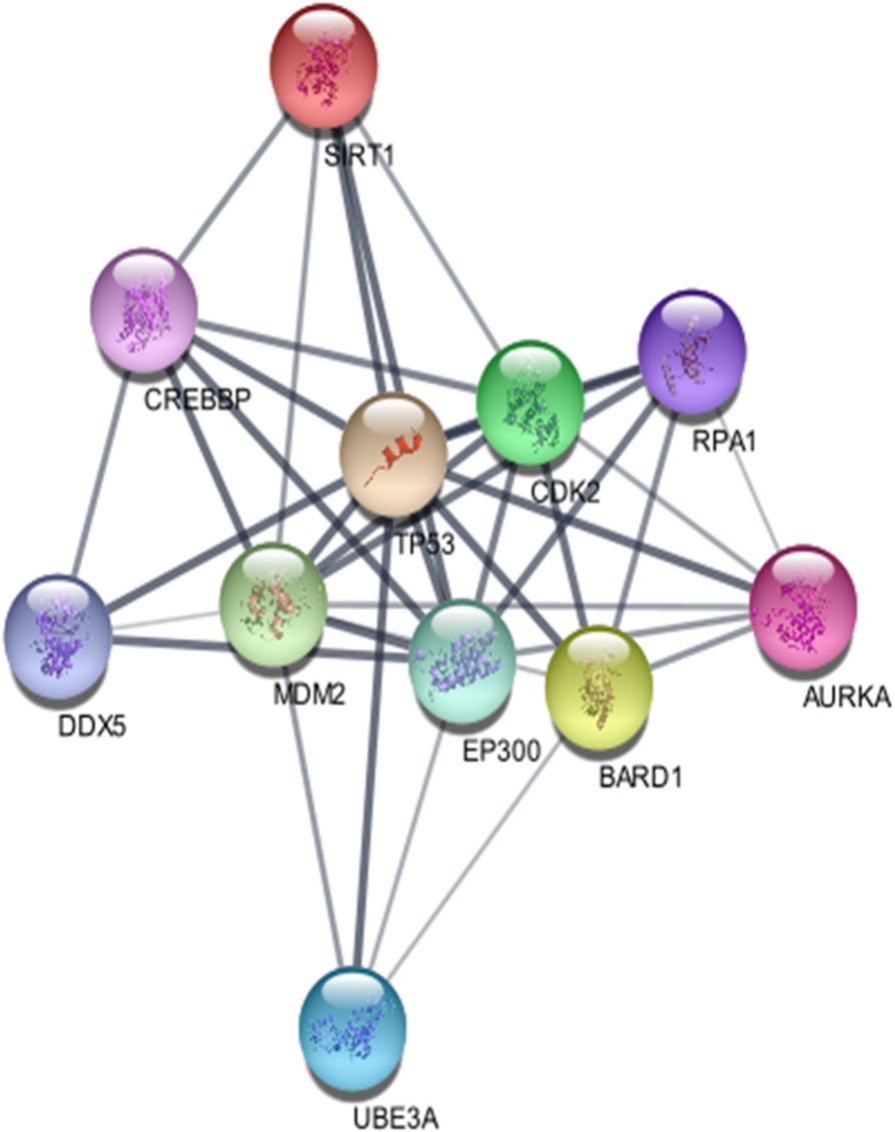
TP53 and its associated genes that as well contribute to breast cancer mutations. CDK2, CREBBP, RPA1, and UBE3A are some of the novel genes

TP53 (tumor protein p53) codes for the cellular tumor antigen p53, which has mutations in 20–40% of all breast carcinomas, depending on tumor size and stage [50]. Because it arises at an early stage of breast cancer development, several TP53 gene variants have been discovered [50] (Fig. 4).

It promotes apoptosis depending on physiological conditions and cell type [34]. It also acts as a trans-activator in cell cycle regulation, inhibiting cell division by regulating a set of genes involved in the process. The long intragenic noncoding RNA p21 initiates lincRNA-p21 and lincRNA-Mkln1 transcription (lincRNA-p21). LincRNA-p21 is involved in transcriptional repression mediated by TP53, which leads to apoptosis. Notch signaling crossover is affected by this protein. CDK7 kinase forms a complex with the CAK complex in response to DNA damage [49]. It suppresses cell cycle progression by reducing CDK7 kinase activity [39]. Some, but not all, TP53-inducible promoters boost isoform 1’s transactivation activity. Isoform 4 reduces isoform 1-mediated growth inhibition by suppressing transactivation activity. Isoform 7 reduces apoptosis caused by isoform 1 by inhibiting CLOCK-ARNTL/BMAL1-mediated PER2 transcriptional activation, which regulates the circadian clock.

### Genes associated with CHEK2 gene

**Fig. 5:**
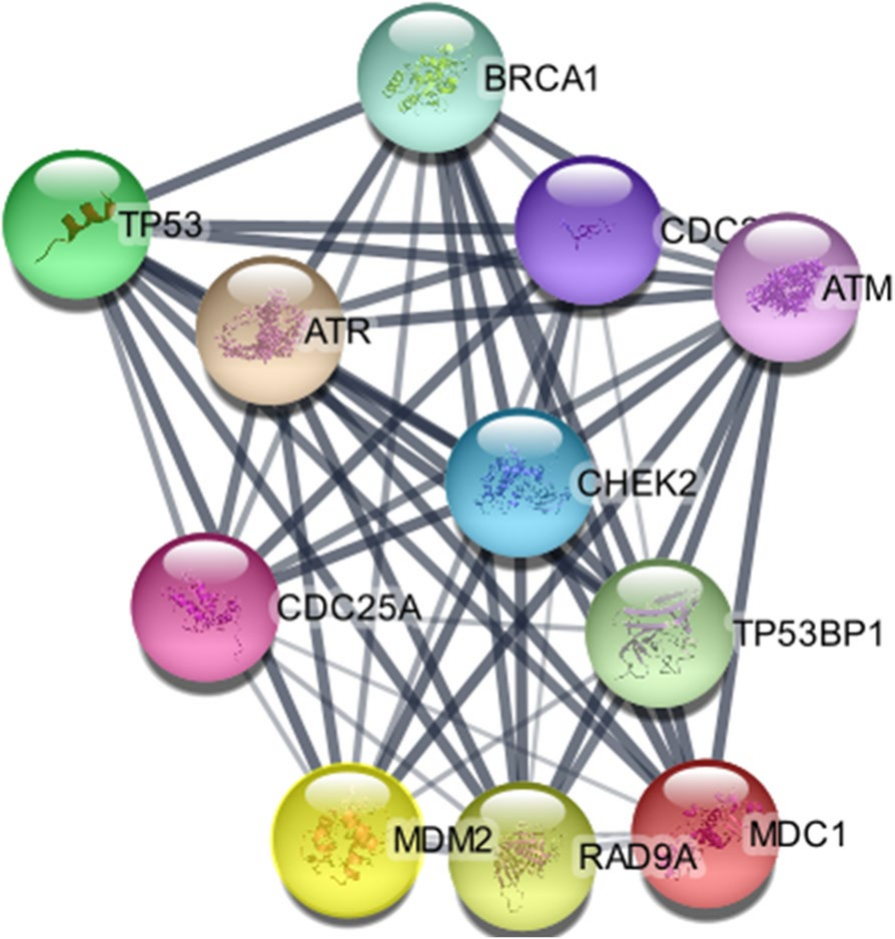
CHEK2 is a tumor suppressor in the body, but it has been related to a range of tumors when it is mutated. It is a relatively severe gene mutation. BRCA1, BRCA2, TP53, ATR, MDM2, and PALB2 have all been linked to a higher risk of breast cancer. RAD9A and CDC25A are the predicted genes

The tumor suppressor gene CHEK2 encodes the protein CHK2, a serine-threonine kinase implicated in DNA repair, cell cycle arrest, and apoptosis in response to DNA damage. CHEK2 gene mutations have been associated to a range of cancers [51] (Fig. 5).

## Discussion

**Fig. 6:**
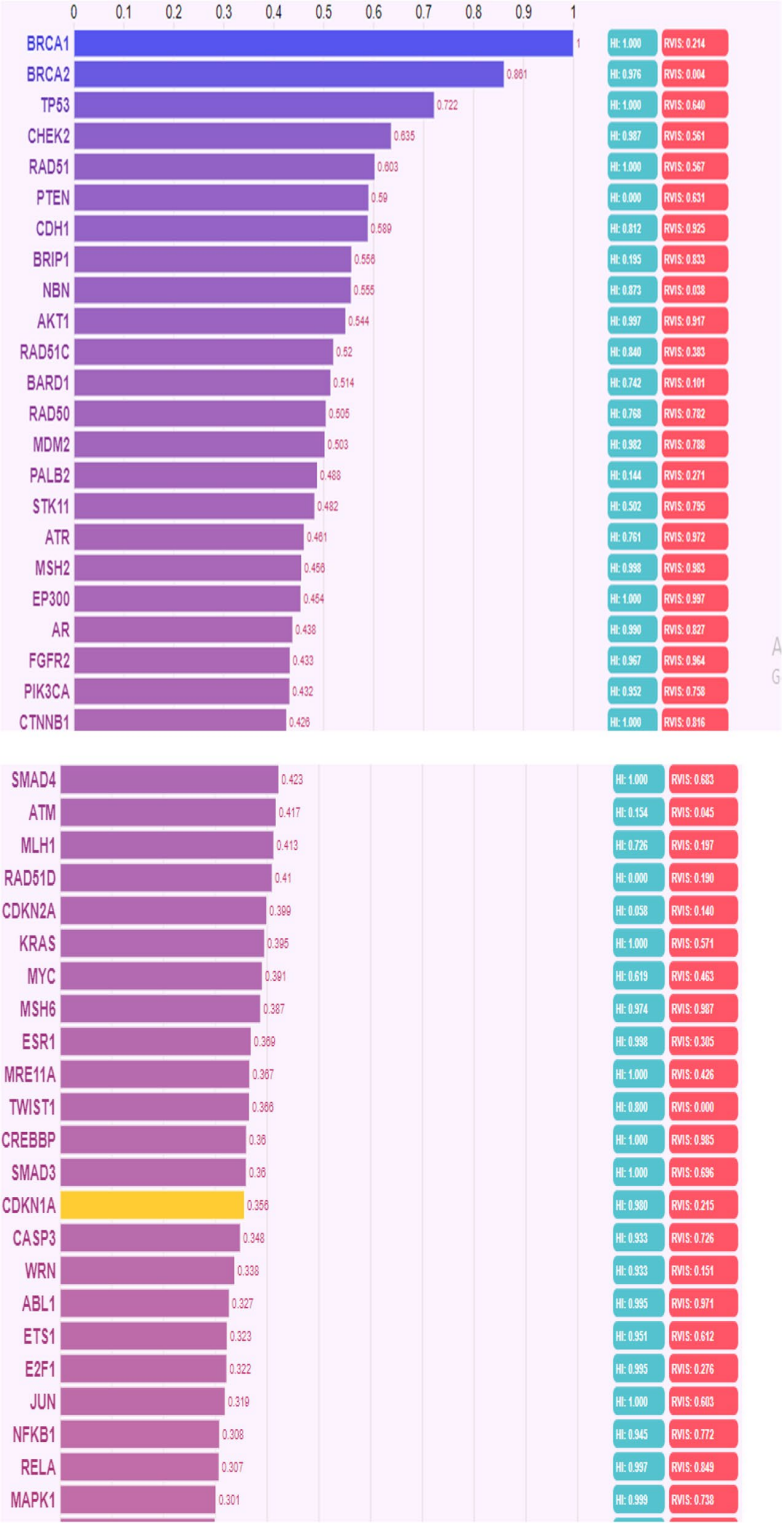
The genes as shown in a barplot with their score values developed using the Phenolyzer platform from Nature Methods at 2020 were prioritized based on their high risk of developing breast cancer

Several genes and pathways linked to breast cancer etiology have been discovered [37]. However, despite these attempts, the processes behind breast cancer pathogenesis have remained unknown [37]. The goal of this research was to build a list of the most important genes that had already been discovered and to find new ones that had not yet been clearly related to breast cancer. This study’s findings may have a wide range of clinical implications [52]. Due to its complexity, breast cancer is frequently caused by mutations in numerous functional genes [10]. Using TM data and gene network information as input, Phenolyzer prioritized breast cancer-related genes by creating a list of 21,921 genes ranked by score. Seed genes and new genes were mined by Maayan Lab’s Geneshotsav 2021 technology for gene mining. Gene seed is the one-of-a-kind genetic substance that can turn a regular person into a superhuman space marine [53]. The anticipated genes are the novel genes. This meets the first goal because the table contains mined genes with their status indicated as novel and anticipated genes. Microarrays and other genetic investigation methods make studying so many genes challenging [37]. Artificial intelligence models were combined to determining gene functioning, hence enabling to overcome the mutational pathways in breast cancer. The most important genes are gathered into a barplot (Fig. 6). The following were the top 14 genes identified by Phenolyzer (with a score of 0.5–1.0).

### Genetic mutations in BRCA1 and BRCA2

The majority of inherited cases of breast cancer are linked to BRCA1 (BReast CAncer Gene One) and BRCA2 (BReast CAncer Gene Two) mutations. The BRCA1 and BRCA2 genes are found in everyone [55–57]. The BRCA genes are responsible for cell damage repair and proper cell proliferation in breast, ovarian, and other tissues. These genes stop functioning when they are altered and passed down through generations, increasing the risk of breast cancer. BRCA1 and BRCA2 mutations are thought to be responsible for up to 10% of all cases of breast cancer or one in every ten. Breast cancer is not always caused by a BRCA1 or BRCA2 mutation. Changes in other areas of SNPs have been related to an increased risk of breast cancer in both BRCA1 mutation carriers and noncarriers, according to studies [57]. Breast cancer is more likely to develop in women who have a BRCA1 or BRCA2 mutation with a family history of breast cancer, ovarian cancer, or other cancers [46, 58]. Despite this, the majority of breast cancer patients do not have a genetic mutation or a family history of the disease [59].

### Mutations in high-risk genes

#### PALB2

It is a partner and localizer of BRCA2, and by binding BRCA2 and RAD51 to DNA breaks, it plays a vital role in homologous recombination repair (HRR). By stabilizing the nucleoprotein filament against a disruptive BRC3-BRC4 polypeptide, RAD51 is able to resist the inhibitory action of replication protein A (RPA). Is there a gene that makes a protein that works with BRCA2 to repair DNA damage and stop tumors from growing? According to research, women with a PALB2 mutation had a 14% probability of having breast cancer by the age of 50, but that risk increases to 35% by the age of 70. Those who have a family history of breast cancer have a 58% probability of getting it before they turn 70 [34]. Women who have a defective BRCA1 gene, on the other hand, have a 50 to 70% probability of having breast cancer by the age of 70. Women who inherited a defective BRCA2 gene had a 40 to 60% chance of having breast cancer by the age of 70.

#### PTEN, tumor suppressor, phosphatase, and tensin homolog

Dephosphorylates phosphorylated proteins on tyrosine, serine, and threonine as a dual-specificity protein phosphatase [49]. It also functions as a lipid phosphatase at the D3 position of the inositol ring, removing the phosphate from phosphatidylinositol. It is involved in cell growth control [49]. Cowden syndrome is a rare disorder in which persons with a defective PTEN gene have an increased risk of developing benign and malignant breast tumors, as well as growths in the digestive tract, thyroid, uterus, and ovaries. PTEN mutant carriers are thought to have a lifetime breast cancer risk of 25 to 50% [60, 61]. According to other research, the risk is between 77 and 85%. At the time of diagnosis, the average age was between 38 and 40 years old.

### Gene variants with a moderate to high risk

ATM (serine/threonine protein kinase) is a DNA damage sensor that activates checkpoint signaling in response to DSBs, apoptosis, and genotoxic stimuli such as ionizing ultraviolet A radiation (UVA) [53]. It is involved in the repair of damaged DNA. In cells, DNA stores genetic information. The brain development disorder ataxia-telangiectasia is caused by inheriting two defective copies of this gene. In some families, inheriting one abnormal ATM gene has been associated with a higher risk of breast and pancreatic cancer. Because the abnormal gene prevents the cells from repairing damaged DNA, this is the case. According to study, ATM mutation carriers had a 33 to 38% lifetime risk of breast cancer (by age 80). A lifetime risk of 69% is estimated for those with a specific sort of mutation affecting a specific location on the ATM gene. The CDH1 gene produces a protein that facilitates tissue formation by allowing cells to interact. A CDH1 gene mutation increases the risk of stomach cancer in children. A person’s chances of developing stomach cancer increase by 83% over their lifetime. Women who carried a CDH1 gene mutation had a 39 to 52% lifetime risk of invasive lobular breast cancer [61].

### Moderately harmful gene mutations

In the presence of DNA double-strand breaks, CHEK2 is required for checkpoint-mediated cell cycle arrest, DNA repair activation, and apoptosis [53]. Gene is responsible for the production of a tumor-suppressing protein. Breast cancer risk is increased by twofold when the CHEK2 gene is defective. It also increases your chances of developing colorectal and prostate cancer. Breast cancer risk is anticipated to be elevated by 10 to 20% in women with CHEK2 mutations and a family history of the disease. The NBN gene is in charge of producing nibrin, a protein that helps cells repair DNA damage. Nijmegen breakage syndrome is caused by a mutation in the NBN gene, which results in stunted growth in children. Nijmegen breakage syndrome is associated with a lower stature and an increased risk of cancer, particularly breast cancer. Despite the absence of proof, studies suggest that people with specific NBN mutations have a two- to three-fold increased risk of developing cancer. Neurofibromatosis type 1 is caused by an NF1 mutation, which raises the incidence of gastrointestinal stromal tumors and central nervous system malignancies, which are tumors that form in the walls of the stomach or intestines. Cancer affects over 60% of people at some point in their lives. According to several studies, women with an NF1 mutation are more likely to develop breast cancer, especially before the age of 50. The STK11 gene controls cell proliferation. Due to a faulty STK11 gene, persons with Peutz-Jeghers syndrome develop a kind of polyp termed a hamartomatous polyp, mostly in the small intestine but also in the stomach and colon. Breast cancer, lung cancer, and ovarian tumors are all more common in those with Peutz-Jeghers syndrome, in addition to gastrointestinal cancers.

### Breast cancer risk is linked to genetic mutations

Further gene changes are occasionally discovered in families with a substantial cancer history. Breast cancer risk may or may not be increased by mutations in the genes mentioned below. More research is needed to determine whether there is an increased risk of breast cancer. BARD1 (BRCA1-associated RING domain 1) is a BRCA1-related DNA-repair gene. BARD1 mutations, according to some study, may increase the risk of breast cancer. BRIP1 is also involved in DNA repair. A BRIP1 mutation has been related to an elevated risk of ovarian cancer in the long run. Through mining done, research shows that it is linked to breast cancer. MLH1, MSH2, MSH6, PMS2, and EPCAM are all mismatch repair genes that correct errors in DNA replication. Lynch syndrome, also known as hereditary nonpolyposis colorectal cancer, is a condition caused by genetic abnormalities (HNPCC). Patients with Lynch syndrome are more prone to develop colon cancer, as well as breast and ovarian cancer [62]. MLH1 and MSH2 mutations have been linked to breast cancer in several studies. The genes RAD51C and RAD51D are involved in the repair of DNA damage. Both have been linked to an increased risk of ovarian cancer over time.

#### RAD52

Double-stranded break repair protein RAD52 homolog promotes the annealing of complementary single-stranded DNA and stimulates the RAD51 recombinase, both of which are essential for genetic recombination and DNA repair.

Understanding the molecular pathways underlying these disorders requires identifying disease-related genes, which is a complex task. Specifically, a group of breast cancer-associated genes was curated from the Geneshotsav 2021 database, and their distribution in an integrated protein–protein interaction network was examined using Cytoscape 3.9.1. The combined score was set to 0.5, resulting in a gene network with 122 nodes and 175 edges, revealing the breast cancer’s complex and diverse nature once more. In this study, the STRING database was used to create a genome-wide gene network using the most recent interaction data from the database, revealing that breast cancer-associated genes are significantly closer to one another than at random, confirming previous findings about disease gene modularity in a PPI network [63].

These genes were distributed non-randomly on the subnetworks of the top breast cancer-associated Geneshot pathways, indicating that these geneshot pathways are activated in a nonuniform manner (see Fig. 1). The nonrandom distribution of breast cancer-associated genes was used to improve an artificial model that built novel cancer genes by combining network reconstruction based on local random walk dynamics with subnetworks spanning many pathways. Because the top predicted genes are more enriched on known breast cancer-associated gene ontologies, this technique outperforms disease gene prediction without integrating information from the Geneshot pathways in inferring breast cancer-associated genes. Finally, a review of the top anticipated novel genes revealed that the vast majority of them have at least wet-lab experiments on cell lines to back them up. Finally, a computational tool for finding novel breast cancer-associated genes was developed, which could be used in future research.

In relation to the stated objective of offering alternative valuable computational approaches to discovering pathways and enriched networks of genes in the detection of breast cancer, this was achieved as demonstrated using Fig. 1, which depicts a gene network, Fig. 2 which depicts the networking of seed genes which are linked previously developed medications.

## Conclusion

The combination of TM and bioinformatics approaches (such as gene recognition, normalization, and ontology) can aid scientists in better understanding of gene networks and pathways, as well as candidate genes and novel genes. Prior to completing in vivo investigations or microarrays, researchers must be able to focus on relevant candidates and unique genes for breast cancer. The top 14 genes associated with breast cancer in the current study were BRCA1, BRCA2, TP53, CHEK2, RAD51, PTEN, CDH1, BRIP1, NBN, AKT1, RAD51C, CASP3, CREBBP, and SMAD3. The findings are consistent with microarray and genome-wide analyses, which is a positive indication.

### Limitations

To increase the practical applicability of such a genome-directed diagnosis, it is necessary to investigate and refine fundamental restrictions these techniques used. For beginners, computer models could only generate breast cancer genes. As the prospective cohort increases, more cancer types and molecularly or histologically different cancer subtypes will be added. Second, the method depended on focused clinical sequencing of known cancer-related genes, but it might be improved if impartial whole-exome or genome sequencing becomes available in clinical practice. Third, while the application to tumors with unknown origins has the most clinical potential, the lack of a definitive histological diagnosis makes benchmarking forecast accuracy and changing therapy options difficult in such patients. Finally, more prospective clinical research is needed to determine the true mutational signatures, as well as the frequency with which this technique resolves difficult diagnostic situations, changes existing diagnoses, and modifies treatment.

## Data Availability

Data for this manuscript can be made available upon request.

## Abbreviations

AI: Artificial intelligence
PPIs: Protein-protein interactions
ERs: Estrogen receptors
BRCA1: BReast CAncer Gene One
BRCA2: BReast CAncer Gene Two
NF1: Neurofibromatosis type 1
TM: Text mining
HRR: Homologous recombination repair
